# Knowledge, Attitude, Practice, and Determinants of Community Health Workers' Involvement Toward NCD Prevention and Control in Northern Tanzania: A Cross‐Sectional Study

**DOI:** 10.1002/hsr2.70978

**Published:** 2025-06-30

**Authors:** Harold L. Mashauri, Cornel M. Angolile, Florida J. Muro

**Affiliations:** ^1^ Department of Internal Medicine KCMC University Moshi Tanzania; ^2^ Department of Epidemiology and Biostatistics, School of Public Health KCMC University Moshi Tanzania; ^3^ Department of Community Health, School of Public Health KCMC University Moshi Tanzania; ^4^ Department of Physiology KCMC University Moshi Tanzania; ^5^ Department of Community Health Kilimanjaro Christian Medical Centre Moshi Tanzania

**Keywords:** community healthcare workers, NCD prevention and control, non‐communicable diseases, public health

## Abstract

**Background and Aim:**

Globally, non‐communicable diseases (NCDs) are the leading cause of preventable morbidity and premature mortality. In sub‐Saharan Africa, NCDs will be the leading cause of mortality by 2030. In 2018, WHO reported that NCDs accounted for about 33% of all deaths in Tanzania. Community health workers (CHWs) are among the key stakeholders in prevention and control of NCDs. In developing countries like Tanzania, CHWs play an extensive role in prevention and control of communicable diseases, however, their simultaneous involvement toward NCDs is limited. This study aimed to determine knowledge, attitude, practice, and determinants toward CHWs' involvement in NCDs prevention and control in northern Tanzania.

**Methods:**

This was a community‐based analytical cross‐sectional study in northern Tanzania enrolling 191 CHWs. Frequencies and percentages, *χ* ^2^, and logistic regression tests were used to summarize categorical variables and determinants of CHWs' involvement toward NCDs prevention and control, respectively, using SPSS.

**Results:**

Majority of participants had good knowledge (92.1%) and favorable attitude (100%). More than half (63.4%) were involved in NCD prevention and control programs of which only 26.7% and 41.4% reported to have involved in NCDs screening and community mobilization programs, respectively. Only 36.1% and 46.1% reported to have access to NCDs screening tools and to have ever attended either formal NCD seminar or training, respectively. Professional training to practice as CHW, frequency of home visits per week, involvement confidence, attendance of formal seminar or training on NCDs, and accessibility of tools for NCDs screening were determinants of CHWs' involvement in NCDs prevention and control.

**Conclusion:**

Despite the vital role of CHWs toward NCDs prevention and control, their engagement in NCDs screening and community mobilization is still low in northern Tanzania. Professional training among in‐service and newly enrolled CHWs, encouragement of weekly home visits, and NCDs' capacity‐building programs in terms of skills and accessibility of screening tools should be implemented among CHWs to enhance their involvement in NCDs prevention and control.

**Abbreviations**
CHWs: community health workers
CVD: cardiovascular disease
HIV: human immunodeficiency virus
LMICs: low‐ and middle‐income countries
NCDs: non‐communicable diseases
TB: tuberculosis
WHO: World Health Organization

## Definition of Key Terms

*Community health workers:* These are paraprofessionals or lay individuals with an in‐depth understanding of the community culture and language, have received standardized job‐related training of a shorter duration than health professionals, and their primary goal is to provide culturally appropriate health services to the community [1].

*Non‐communicable diseases:* These are chronic health conditions that are not contagious to others. They include cancer, diabetes, cardiovascular diseases, and chronic lung illnesses among others [2].

*NCDs prevention and control practice:* This refers to the involvement in activities which aim to prevent or control different non‐communicable diseases in the community. It includes either screening, health promotion programs like community education, patient referral, or patient management and monitoring (operational definition).

*Premature death:* This refers to the death that occurs before the average age of death in a certain population [3].

## 1 Introduction

The rising burden of non‐communicable diseases (NCDs) and associated mortality is of public health concern, especially in low‐ and middle‐income countries (LMICs) [4, 5]. Globally, NCDs are the leading cause of premature mortality and morbidity, even though they can be significantly prevented [6]. The World Health Organization (WHO) outlined four major NCDs as the leading causes of mortality: cardiovascular diseases (CVD), diabetes mellitus, chronic respiratory diseases, and cancers [5]. These four diseases have four potential modifiable risk factors, namely, unhealthy diet, physical inactivity, harmful alcohol use, and tobacco use, that contribute to over 80% of NCDs' premature mortality [5, 6]. Globally, about 70% of all deaths are due to NCDs, and among them, 87% occur in LMICs [7]. By reducing or eliminating the four modifiable shared risk factors, up to 80% of CVD, type 2 diabetes mellitus, and over one‐third of cancers can be prevented [8]. Almost 36 million people die every year (63% of the global deaths) because of NCDs: of these, 14 million deaths are premature. LMICs contribute up to 86% of the global NCDs' premature deaths [6].

In sub‐Saharan Africa, NCDs will be the leading cause of mortality by the year 2030 [9]. In Tanzania, all NCDs caused about 31% of premature deaths in the year 2012: the four main NCDs caused 16% of the deaths [10]. The cases of NCDs in Tanzania have been increasing abruptly to the extent of needing an immediate action [11]. According to the WHO, NCDs countries' profiles report of 2018, it was estimated that NCDs account for about 33% of all deaths in Tanzania [12]. Moreover, NCDs pose a catastrophic economic burden in Tanzania [13, 14]. While Community Health Workers (CHWs) may play a pivotal role in the prevention and control of NCDs, it is unfortunate that both the national NCDs Strategic Plan of 2008–2018 and 2016–2020 in Tanzania did not clearly indicate their role [15, 16]. Of note, the current Tanzania National NCD Strategic Plan of 2021–2026 embraces a multisectoral collaboration approach with the development of NCD training manuals for primary healthcare facilities [17].

CHWs have been identified as an important part of the public health workforce globally and have been mobilized to provide primary healthcare at the community and household levels [18]. These are trained individuals that assist in providing health promotion, prevention, community empowerment, administering basic curative health services, and linking local communities with health facilities within their locality with the aim being to reduce morbidity and mortality [19]. Despite their potential role in the healthcare system, their role and contributions in many countries, including Tanzania, are mostly limited to maternal and child health like their participation in the immunization of vaccine preventable diseases as well as prevention and treatment of infectious diseases like TB and HIV while majority of their tasks are highly fragmented as their program driven [20]. CHWs are mostly trained to fill the health provider gaps in health systems and healthcare delivery, especially in developing countries due to the shortage of healthcare workers which is more vivid at the primary healthcare level like in Tanzania, whereby HRH gap is at 52% of the needed staffing level by 2021. In developing countries like Tanzania, addressing the recognized major NCDs such as CVD, cancer, diabetes, and chronic respiratory diseases is necessary at the primary care level considering the rising burden of morbidity and mortality they are causing [7, 21]. Through this, different programs and action plans for NCDs are therefore being strengthened for primary prevention and control, and CHWs have the potential to offer support in the provision of primary healthcare services [22, 23]. To address the gaps in human resources for health regarding NCDs, the mobilization of CHWs shows to have promising results [24, 25]. Evidence suggests various roles like community mobilization and screening, and the relevance of CHWs in the management of different NCDs [26].

Given the pivotal role which CHWs can play in prevention and control of NCDs in their communities, a 2017 study in Uganda showed that most of them had little awareness about most NCDs, as a result, this hindered their effectiveness [27]. Another study of 2022 in Uganda reported that the majority of CHWs (75.3%) had general awareness about different NCDs [28]. Moreover, more than half of the study participants reported being involved in NCDs activities like community mobilization in their communities. Several studies have shown that the majority of CHWs had significant knowledge on prevention and control measures toward some NCDs [28, 29]. Importantly, most CHWs admitted the fact that NCDs burden is a significant public health problem and they have a key role to play in prevention and control [27, 28].

Despite the fact that there are limited studies on the proportion of CHWs engaging in the practice of NCDs prevention and control in their communities, the study in Uganda reported that 63.1% of CHWs were involved in NCDs activities even though only few of them participated in community mobilization (20.9%) and referral of patients (20.6%) [28]. Another study reported that more than half of the participants were involved in community education and counseling on cervical cancer among all NCDs [29].

In one study, CHWs reported that lack of knowledge, lack of training, and negative community perception of NCDs among community members as challenges which prevent them from being effective in addressing NCDs in their communities even though another study reported that lack of financial remuneration was not a barrier in providing NCDs‐related services [27, 28]. Some studies pointed contrary that financial remuneration plays a role in CHWs participation in fighting NCDs [30, 31].

Despite other countries like India having established the role of CHWs in NCDs prevention and control, the role of CHWs in delivering primary prevention and control interventions for NCDs in Tanzania is limited [32, 33, 34]. Most CHWs are willing and wish to participate in different NCDs intervention programs but do not have knowledge on NCDs to enable them offer required preventive and control measures despite easy availability of online WHO community‐based guidelines resources which includes prevention and control of NCDs [WHO Package for Essential Non‐communicable (PEN) diseases interventions for primary healthcare] [35, 36]. Moreover, evidence suggests that CHWs are interested in delivering NCDs' prevention services within their communities and thus it is crucial to understand their level of knowledge, attitudes, and perceived role in addressing NCDs as part of the feasibility of involving them in the prevention and control of these diseases [37, 38]. Information on the knowledge and attitudes of CHWs regarding NCDs would provide insights into the competencies and training needs for them to perform their tasks effectively. This study will assess the involvement of CHWs in the prevention and control of NCDs in northern Tanzania with a focus on their knowledge, attitudes and practices, and finding out related determinants. Results will enable policymakers and community health‐related stakeholders to formulate and implement informed interventions in providing CHWs with a conducive and enabling environment toward the prevention and control of NCDs in their communities.

## 2 Materials and Methodology

The reporting of this study has followed the STROBE guideline [39].

### 2.1 Study Design, Setting, and Population

This was a community‐based analytical cross‐sectional study conducted from May 2023 to September 2023 among 191 CHWs in northern Tanzania, specifically from Moshi Municipal in Kilimanjaro and Arusha Municipal in Arusha. Moshi and Arusha Municipals are urban districts located in northern Tanzania. They were purposively selected due to the presence of the most active CHWs‐based programs in northern Tanzania. The eligibility criteria were: a participant who was 18 years or older during the time of the study, registered and assigned as a CHW in a study area. The exclusion criteria were: CHWs who have retired or not active.

Moshi Municipal and Arusha Municipal have 90 and 154 registered CHWs, respectively, making a total of 244. Based on the total number of CHWs in the study area, the sample size was calculated using Epi‐Info statistical software and the minimum required sample size was 150. Convenience sampling technique was used to enroll participants in the study.

### 2.2 Data Collection Methods and Tools

An interviewer‐administered questionnaire was used for data collection adapted from previous studies with already validated tools [28, 29]. The questionnaire was both in English and Swahili languages. It was tested and modified with a series of consultation from NCD and community health experts prior to actual data collection.

Trained research assistants administered the questions using an electronic tool (KOBO) with a total of 52 questions. The first section comprised questions on demographic characteristics of participants such as age, gender, level of education, and social economic status (employment); the second section assessed knowledge of CHWs on NCDs, including whether they can correctly define NCDs or know examples of such diseases, as well as if they think these diseases can be prevented and/or treated, and methods of prevention and control. Specific knowledge on causes, risk factors, and complications of different NCDs was also assessed in this section. The third section explored the attitudes of the CHWs toward NCDs and their burden in Tanzania. This was assessed using a Likert scale (*Strongly agree*, *Agree*, *Neutral*, *Strongly disagree*, and *Disagree*). The fourth section assessed the current involvement of CHWs in the prevention and control of NCDs. This was determined in an aspect of whether they have been involved in NCD screening, patient management, palliative care, and/or community mobilization. The level of confidence in their involvement in these activities was also assessed in this section using a Likert scale (*Not confident at all*, *A little confident*, *Moderately confident*, *Confident*, *Very confident*). The fifth section intended to assess on the possible factors that contributes either positively or negatively to the CHWs' involvement in NCDs prevention and control in their communities like availability of tools for NCD screening, including challenges/barriers faced based on Likert scale ranging *Strongly disagree* to *Strongly agree* in the following areas: low NCD knowledge, lack of training on NCDs, poor community perception toward NCDs, and lack of support from health workers/nearby health facility.

### 2.3 Study Variables

The main outcomes of interest were knowledge score, favorable attitude, and practices/involvement of CHWs in NCD prevention and control. Knowledge score was determined from the number of questions answered correctly out of the total knowledge questions. More than 50% of the questions answered correctly were translated as satisfactory knowledge. Moreover, perceived knowledge on five major NCDs was also assessed by five questions on a 4‐point Likert scale where the responses, *I don't know*, *I know a little*, *I know some*, and *I know a lot*, had scores 0 through 3, respectively. The highest possible score was 15, the lowest being 0. Therefore, good perception was defined by a score of more than 50% of the highest score.

For attitude, a 5‐point Likert scale was used for a total of six questions of which the score ranging from 1 for *Strongly disagree* to 5 for *Strongly agree*, thus the highest possible attitude score was 30, the lowest being 6. Attitude score more than 50% of the highest possible score was regarded as favorable attitude.

Practice (involvement of CHWs on NCD prevention and control) was determined by four major variables, which were CHWs involvement in “NCD screening,” “patient management,” “patient care,” and “community mobilization.” Of these, community mobilization was ascertained from nine different questions regarding the CHWs' frequency of discussing the common NCDs and their risk factors which were on a 5‐point Likert scale where the responses *Never* and *Always* were given a score 1 and 5, respectively. The highest possible score was 45. The participant's score of more than 50% of the total was regarded as good health mobilization. The average of the four variables was then used to elicit the involvement of CHWs in the NCD prevention and control where being involved in two or more activities stated was translated as good practice.

### 2.4 Data Analysis

Data cleaning and analysis were performed using the SPSS, 20th version. Frequencies and percentages were used to summarize categorical variables while means/medians and standard deviations/Interquartile range for numerical variables. The two‐sided *χ* ^2^ test was used to determine factors associated with CHWs' involvement in NCDs prevention and control with statistical significance at *p* < 0.05 after which logistic regression estimated the odds ratio (OR) with the 95% confidence interval.

### 2.5 Ethical Approval and Consent to Participate

Ethical approval was sought from Kilimanjaro Christian Medical University College Research and Ethics Review Committee (KCMUCo‐CRERC) with certificate registration number 2610. Permission letters to conduct a study were obtained from local government authorities in Moshi and Arusha Municipals. Moreover, written informed consent was obtained from each participant after explaining to them and reading the study information prior to start filling the survey so that they can make an informed decision to participate or withdraw from the study without losing their dignity. The consent information contained a description of the study purpose, risk and benefits of participation, nature of participant involvement, and confidentiality. Also, the privacy and safety of the participants' information were considered during the study by using a unique identification number instead of their names. Participation in this study was voluntary and participants were allowed to terminate their participation or not responding to a certain question willingly.

## 3 Results

### 3.1 Participants' Background Characteristics

A total of 191 CHWs met the inclusion criteria and were enrolled. Their mean age was 45 ± 12 years. Majority (146, 76.4%) were females and 115 (60.2%) were practicing in Arusha. Over half (112, 58.6%) had a working experience of < 10 years as CHW and the vast majority (177, 92.7%) admitted to have received a special training as CHW mostly by physicians (96, 50.3%) (Table 1).

**Table 1:** Sociodemographic characteristics of study participants (*N* = 191).

Variable	*n*	%
Sex		
Male	45	23.6
Female	146	76.4
Age (years)		
Mean (SD)	45.04 (12.16)	
< 25	8	4.2
25–34	37	19.4
35–44	36	18.8
45+	110	57.6
Area of practice		
Kilimanjaro	76	39.8
Arusha	115	60.2
Education level		
Primary education	91	47.6
Secondary education	69	36.1
Higher education	31	16.2
Working experience as CHW		
< 10 years	112	58.6
10+ years	79	41.4
Any special training as CHW?		
Yes	177	92.7
No	14	7.3
Home visits per week		
< 3	90	47.1
3+	101	52.9
Frequency of trainings from health professionals		
At least once a week	10	5.2
At least once a month	23	12.0
At least once every 2 months	145	75.9
At least once every 6 months	13	6.8
Group of health professionals from whom you receive formal teachings		
Physicians	96	50.3
Nurses	17	8.9
Medical residents/students	2	1.0
Other CHWs	47	24.6
Other educators	29	15.2
Occupation		
Not employed	39	20.4
Self‐employed	143	74.9
Employed	9	4.7

### 3.2 CHWs' Knowledge on NCDs' Prevention and Control

Majority of the CHWs (117, 89.7%) defined correctly what NCDs are. Most CHWs stated the examples of NCDs including diabetes (97.9%), hypertension (95.3%), and stroke (84.3%). In total, 99% of CHWs agreed that smoking affects person's health, however, only a few (7.3%) knew the cut‐off points for overweight BMI, and 27.7% knew the recommended time for moderate‐intensity physical activity per week. Knowledge on hypertension, diabetes, chronic obstructive pulmonary diseases (COPD), and cancer screening was also assessed. Overall, 92.1% had satisfactory knowledge scores on NCDs prevention and control (Table 2). Also, perceived general knowledge on different NCDs was assessed with most CHWs (60.2%) having poor perception on their knowledge of the medical conditions (Table 3).

**Table 2:** CHWs' knowledge about NCDs (*N* = 191).

Variable	Category	*n*	%
NCD cannot be spread between people	True	117	89.5
	False	15	7.5
	I don't know	5	2.6
Examples of NCDs	Diabetes	187	97.9
	Tuberculosis	5	2.6
	Asthma	141	73.8
	Stroke	162	84.8
	AIDS	4	2.1
	Cancer	161	84.3
	Malaria	93	48.7
	Hypertension	182	95.3
	I don't know	2	1.0
Active smoking affects a person's health	True	189	99
	False	1	0.5
	I don't know	1	0.5
Smoking around others does not harm their health	True	141	73.8
	False	49	25.7
	I don't know	1	0.5
Smoking affects lungs	True	190	99.5
	False	0	0
	I don't know	1	0.5
Smoking affects the heart	True	133	69.6
	False	24	12.6
	I don't know	34	17.8
Source with largest amount of salt	The table salt added to food	173	90.6
	The salt in milk, meat, and vegetables	0	0
	The salt in factory‐made foods like bread, sausages, and canned foods	9	4.7
	I don't know	9	4.7
Which BMI is considered as being overweight	Correct response (25–29.9)	14	7.3
	Incorrect response	46	24.1
	I don't know	131	68.6
Total time of moderate‐intensity physical activity per week	Correct response (150 min/week)	53	27.7
	Incorrect response	81	42.4
	I don't know	57	29.8
Risky diet for NCDs	Junk/processed foods	169	88.5
	Natural foods	9	4.7
	I don't know	13	6.8
*Knowledge on high blood pressure and risk factors*			
Effect of salt on blood pressure	Correct response (raises blood pressure)	160	83.8
	Incorrect response	9	4.7
	I don't know	22	11.5
*High blood pressure affects the following body parts*			
The brain	Yes	140	73.3
	No	20	10.5
	I don't know	31	16.2
The kidneys	Yes	108	56.5
	No	31	16.2
	I don't know	52	27.2
The stomach	Yes	27	14.1
	No	84	44
	I don't know	80	41.9
The heart	Yes	168	88
	No	8	4.2
	I don't know	15	7.9
People with high blood pressure are more likely to have stroke	True	167	87.4
	False	4	2.1
	I don't know	20	10.5
Cardiovascular diseases risk factors	Insomnia	146	76.4
	Running	48	25.1
	Stress	177	92.7
	Older age	141	73.8
	Higher BMI	182	95.3
	Vegetarian diet	5	2.6
	Smoking	142	74.3
*Knowledge on diabetes*			
Diabetes is when there is chronically high blood sugar	True	157	82.2
	False	13	6.8
	I don't know	21	11
Signs and symptoms of diabetes type 2	Frequent urination	180	94.2
	Increased thirst	145	75.9
	Loss of appetite	103	53.9
	Weight loss	160	83.8
	Fatigue	171	89.5
	I don't know	9	4.5
The following is not a complication of Diabetes	Damage to the heart	106	55.5
	Loss of vision	140	73.3
	Loss of memory	131	68.6
	Loss of sensation to the feet	135	70.7
	Damage to the kidneys	122	63.9
	Erectile dysfunction	133	69.6
	Poor healing of wounds	138	72.3
	I don't know	12	6.3
*Knowledge on COPD*			
Causes of COPD	Air pollution	167	87.4
	Chronic asthma	145	75.9
	Genetics	123	64.4
	Smoking	172	90.1
	Recurrent lung infections	128	67
	I don't know	13	6.8
Signs and symptoms of COPD	Cough	174	81.1
	Leg swelling	109	57.1
	Shortness of breath	175	91.6
	Fatigue	160	83.8
	Sputum production	160	83.8
	I don't know	12	6.3
*Knowledge on cancer screening*			
Medical condition screened with pap test	Sexually transmitted infections	9	4.7
	Cervical cancer	40	20.9
	Ovarian cancer	7	3.7
	Pregnancy	3	1.6
	Syphilis	6	3.1
	I don't know	150	78.5
When to undergo a pap test for asymptomatic average‐risk women	Correct response (21 years)	25	13.1
	Incorrect response	19	9.9
	I don't know	147	77
How often should women who had two normal pap tests continue having the test?	Correct response (2–3 years)	8	4.2
	Incorrect response	32	16.8
	I don't know	151	79.1
Can cervical cancer be prevented by vaccine?	Yes	156	81.7
	No	14	7.3
	I don't know	21	11
At what age should a woman start screening for breast cancer regularly?	Correct response (30–40 years)	39	20.4
	Incorrect response	109	57.1
	I don't know	43	22.5
At what age should a man start screening for prostate cancer?	Correct response (above 45)	85	44.5
	Incorrect response	56	29.3
	I don't know	50	26.2
Overall NCDs knowledge	Satisfactory Knowledge	176	92.1
	Poor Knowledge	15	7.9

**Table 3:** Perceived CHWs' knowledge on different NCDs (*N* = 191).

Variable	Category	*n*	%
High blood pressure	I know a little	99	51.8
	I know some	50	26.2
	I know a lot	30	15.7
	I don't know	12	6.3
Myocardial infarction	I know a little	88	46.1
	I know some	45	23.6
	I know a lot	12	6.3
	I don't know	46	24.1
Stroke	I know a little	82	42.9
	I know some	39	20.4
	I know a lot	29	15.2
	I don't know	41	21.5
Diabetes	I know a little	87	45.5
	I know some	38	19.9
	I know a lot	56	29.3
	I don't know	10	5.2
Chronic obstructive pulmonary diseases (COPD)	I know a little	81	42.2
	I know some	46	24.1
	I know a lot	25	13.1
	I don't know	39	20.4
General perceived knowledge of NCDs	Good Perception	76	39.8
	Poor Perception	115	60.2

### 3.3 CHWS' Attitudes Toward NCD Burden, Prevention, and Control

Majority (152, 79.6%) of the CHWs agreed that NCDs are now common among Tanzanians including CVDs, diabetes, and cancer. In total, 99.5% of the CHWs agreed that early detection of NCDs plays an important role in their prevention and control. Most of the CHWs (97.9%) agreed that they have a very important role to play in NCDs' prevention and control in their communities (Table 4). Generally, all CHWs (100%) had favorable attitude.

**Table 4:** CHWs' attitudes toward NCD burden, prevention, and control (*N* = 191).

Statement	Strongly agree (%)	Agree (%)	Neutral (%)	Disagree (%)	Strongly disagree (%)
NCDs are common among Tanzanians	58 (30.4)	94 (49.2)	8 (4.2)	28 (14.7)	3 (1.6)
Cardiovascular diseases are becoming more common in Tanzania	64 (33.5)	117 (61.3)	4 (2.1)	6 (3.1)	0 (0)
Diabetes is becoming more common in Tanzania	75 (39.3)	108 (56.5)	4 (2.1)	4 (2.1)	0 (0)
Cancer is becoming more common in Tanzania in general	77 (40.3)	99 (51.8)	7 (3.7)	7 (3.7)	1 (0.5)
Early detection plays an important role in NCDs prevention and control	94 (49.2)	96 (50.3)	1 (0.5)	0 (0)	0 (0)
As a community health worker, I have a very important role to play in NCDs prevention and control in my community	71 (37.2)	116 (60.7)	4 (2.1)	0 (0)	0 (0)

Attitudes toward NCDs burden, prevention, and control		***n***		**%**	
Favorable attitude		191		100	
Unfavorable attitude		0		0	

**Figure 1:**
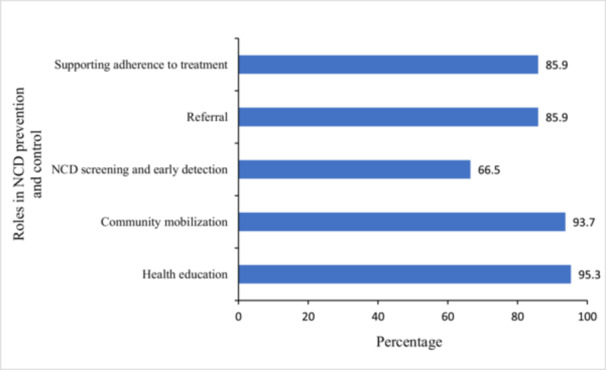
Roles a CHW can play in the prevention and control of NCDs.

Figure 1 highlights the roles which CHWs identified they can play in prevention and control of NCDs. The vast majority (95.3% and 93.7%) identified that they have a role to play in providing health education and community mobilization, respectively, while 66.5% believed they had a role to play in NCD screening and early detection.

### 3.4 CHWs' Practice Involvement, Enabling Factors, and Challenges Toward NCDs Prevention and Control

Overall, 63.4% of the CHWs were involved in at least one of the activities related to NCDs prevention and control. The activities include NCD screening (26.7%), patient management (12.0%), patient care (41.9%), and health mobilization whereby 41.4% had good mobilization. Community health mobilization was ascertained from the questions regarding their frequency of discussing NCD‐related topics (responses are depicted in the table) (Table 5).

**Table 5:** CHWs' practice involvement, enabling factors, and challenges toward NCDs prevention and control (*N* = 191).

Variable	Categories	*n*	%
NCDs prevention and control activities involved	NCDs screening	51	26.7
	Patient management	23	12.0
	Patient care	80	41.9
*Frequency of discussing NCD‐related topics in your community*			
Harms of smoking	Never	7	3.7
	Rarely	29	15.2
	Sometimes	20	10.5
	Often	93	48.7
	Always	42	22
Harms of alcohol	Never	9	4.7
	Rarely	20	10.5
	Sometimes	16	8.4
	Often	91	47.6
	Always	55	28.8
Healthy nutrition	Never	4	2.1
	Rarely	16	8.4
	Sometimes	30	15.7
	Often	78	40.8
	Always	63	33
Physical activity	Never	6	3.1
	Rarely	26	13.6
	Sometimes	26	13.6
	Often	82	42.9
	Always	51	26.7
Weight control	Never	7	3.7
	Rarely	30	15.7
	Sometimes	28	14.7
	Often	84	44
	Always	42	22
Cervical cancer screening	Never	36	18.8
	Rarely	27	14.1
	Sometimes	18	9.4
	Often	67	35.1
	Always	43	22.5
Breast cancer screening	Never	49	25.7
	Rarely	39	20.4
	Sometimes	16	8.4
	Often	59	30.9
	Always	28	14.7
Prostate cancer screening	Never	67	35.1
	Rarely	60	31.4
	Sometimes	20	10.5
	Often	35	18.3
	Always	9	4.7
Colon cancer	Never	133	69.6
	Rarely	28	14.7
	Sometimes	10	5.2
	Often	9	4.7
	Always	11	5.8
General mobilization	Good Mobilization	79	41.4
	Poor Mobilization	112	58.6
Involvement in NCDs prevention and control	Yes	121	63.4
	No	70	36.6
*Level of confidence in accurately doing the following as a CHW*			
NCD screening	Not confident at all	42	22
	A little confident	21	11
	Moderately confident	33	17.3
	Confident	66	34.6
	Very confident	29	15.2
Patient management	Not confident at all	88	46.1
	A little confident	20	10.5
	Moderately confident	23	12
	Confident	40	20.9
	Very confident	20	10.5
Patient care (including palliative care)	Not confident at all	48	25.1
	A little confident	17	8.9
	Moderately confident	25	13.1
	Confident	62	32.5
	Very confident	39	20.4
*Level of confidence in accurately counseling community members about the following topics*			
Harms of smoking	Not confident at all	3	1.6
	A little confident	8	4.2
	Moderately confident	32	16.8
	Confident	82	42.9
	Very confident	66	34.6
Harms of alcohol	Not confident at all	4	2.1
	A little confident	9	4.7
	Moderately confident	25	13.1
	Confident	84	44
	Very confident	69	36.1
Healthy nutrition	Not confident at all	4	2.1
	A little confident	11	5.8
	Moderately confident	22	11.5
	Confident	80	41.9
	Very confident	74	38.7
Physical activity	Not confident at all	6	3.1
	A little confident	10	5.2
	Moderately confident	25	13.1
	Confident	87	45.5
	Very confident	63	33
Weight control	Not confident at all	7	3.7
	A little confident	16	8.4
	Moderately confident	24	12.6
	Confident	86	45
	Very confident	58	30.4
Cervical cancer screening	Not confident at all	36	18.8
	A little confident	16	8.4
	Moderately confident	24	12.6
	Confident	86	45
	Very confident	58	30.4
Breast cancer screening	Not confident at all	55	28.8
	A little confident	23	12
	Moderately confident	29	15.2
	Confident	45	23.6
	Very confident	39	20.4
Prostate cancer screening	Not confident at all	82	42.9
	A little confident	26	13.6
	Moderately confident	23	12
	Confident	40	20.9
	Very confident	20	10.5
Colon cancer	Not confident at all	123	64.4
	A little confident	23	12
	Moderately confident	17	8.9
	Confident	17	8.9
	Very confident	11	5.8
General confidence in involvement toward NCD prevention and control	High Confidence	133	69.6
	Low Confidence	58	30.4
*Enabling factors*			
Ever attended a seminar or formal training on NCDs	Yes	88	46.1
	No	103	53.9
Years Past NCDs' Seminar or formal training.	< 2	45	51.1
	2+	43	48.9
*Accessibility of tools/materials for any of the following*			
NCD Screening	Yes	69	36.1
	No	122	63.9
Patient Management	Yes	381	19.9
	No	153	80.1
Palliative Care	Yes	92	48.2
	No	99	51.8
*Challenges faced in your involvement in different activities in your community toward NCDs prevention and control*			
Lack/insufficient knowledge on NCDs prevention and control	Yes	161	84.3
	No	30	15.7
Poor support from health providers (health facilities)	Yes	99	51.8
	No	92	48.2
Lack of good remuneration	Yes	148	77.5
	No	43	22.5
Negative community perception on NCDs	Yes	129	67.5
	No	62	32.5
Lack of community trust in CHWs skills on NCDs prevention and control	Yes	96	50.3
	No	95	49.7

CHWs' level of confidence in their involvement in these activities was also assessed by 12 different questions on a 5‐point Likert scale where the majority (69.6%) had high confidence (determined by those with a score of more than 50% of the total score) (Table 5).

In total, 53.9% of the CHWs had ever attended a seminar or formal training on NCDs with half of them (51.1%) attending within the last year. Majority of the CHWs experienced difficulty in accessing tools to facilitate their involvement in the NCD prevention and control activities with only 36.1% having access to tools for NCD screening (Table 5).

Among many challenges the CHWs face in their involvement in different activities of NCD prevention and control, majority identified insufficient knowledge on NCDs' prevention and control (84.3%) and lack of good remuneration (77.5%) as two major challenges (Table 5).

### 3.5 Determinants of the CHWs' Involvement in NCDs Control and Prevention Activities

CHWs' involvement on NCD prevention and control was significantly associated with whether they have attended any special training (*p* < 0.001), home visits per week (*p* = 0.003), general confidence toward their involvement in NCD prevention and control (*p* < 0.001), attendance on a seminar or formal training (*p* < 0.001), and accessibility for tools of NCD screening (*p* < 0.001) (Table 6).

**Table 6:** Determinants of the CHWs' involvement in NCDs control and prevention activities (*N* = 191).

Variable	Practice		*p*	COR (95% CI)	*p*
	Yes (%)	No (%)			
Sex			0.22		
Male	32 (71.1)	13 (28.9)			
Female	89 (61.0)	57 (39.0)			
Age			0.64		
< 25	4 (50)	4 (50)			
25–34	21 (56.8)	16 (43.2)			
35–44	24 (66.7)	12 (33.3)			
45+	72 (65.5)	38 (34.5)			
Education level			0.59		
Primary education	55 (60.4)	36 (39.6)			
Secondary education	47 (68.1)	22 (31.9)			
Higher education	19 (61.3)	12 (38.7)			
Working experience as CHW			0.55		
< 10	69 (61.6)	43 (38.4)			
10+	52 (65.8)	27 (34.8)			
Any special training as CHW?			< 0.001*		
Yes	119 (67.2)	58 (32.8)		12.31 (2.67–56.82)	0.001*
No	2 (14.3)	12 (85.7)		1	
Home visits per week			0.003*		
< 3	47 (52.2)	43 (47.8)		1	
3+	74 (73.3)	27 (26.7)		2.51 (1.37–4.59)	0.003*
Occupation			0.57		
Not employed	23 (59)	16 (41)			
Self‐employed	91 (63.6)	52 (36.4)			
Employed	7 (77.8)	2 (22.2)			
Overall knowledge			0.05		
Good Knowledge	115 (65.3)	61 (34.7)			
Poor Knowledge	6 (40)	9 (60)			
General perceived knowledge			0.14		
Good Perception	53 (69.7)	23 (30.3)			
Poor Perception	68 (59.1)	47 (40.9)			
General confidence toward NCD prevention and control			< 0.001*		
High confidence	107 (80.5)	26 (19.5)		12.93 (6.28–27.07)	< 0.001*
Low confidence	14 (24.1)	44 (75.9)		1	
Seminar/formal training on NCD			< 0.001*		
Yes	71 (80.7)	17 (19.3)		4.43 (2.3–8.53)	< 0.001*
No	50 (48.5)	53 (51.5)		1	
Accessibility of tools for NCD screening			< 0.001*		
Yes	64 (92.8)	5 (7.2)		14.6 (5.5–38.8)	< 0.001*
No	57 (46.7)	65 (53.3)		1	
*Challenges faced in your involvement in different activities in your community toward NCDs prevention and control*					
Lack/insufficient of knowledge on NCDs prevention and control					
Yes	105 (65.2)	56 (34.8)			
No	16 (53.3)	14 (46.7)	0.22		
Poor support from health providers (health facilities)					
Yes	61 (61.6)	38 (38.4)			
No	60 (65.2)	32 (34.8)	0.61		
Lack of good remuneration					
Yes	92 (62.2)	56 (37.8)			
No	29 (67.4)	14 (32.6)	0.53		

Abbreviations: COR = crude odds ratio; CI = confidence interval.

* *p* < 0.05.

In crude analysis, CHWs who received special training were 12 times more likely to be involved in NCD prevention and control activities (OR = 12.31, 95% CI 2.67, 56.82); those who make more than two home visits per week were 3 times as likely to be involved (OR = 2.51, 95% CI 1.37, 4.59); those with high confidence toward their involvement also were almost 13 times likely of being involved in NCD prevention and control activities (OR = 12.93, 95% CI 6.28, 27.07). Furthermore, those who attended any seminar or formal training on NCD were 4 times more likely to be involved in NCD prevention and control activities (OR = 4.43, 95% CI 2.3, 8.53); and those who had access to tools for NCD screening were almost 15 times as likely to be involved (OR = 14.6, 95% CI 5.5, 38.8) (Table 6).

## 4 Discussion

The aim of this study was to determine knowledge, attitude, practice, and determinants toward CHWs' involvement in NCDs prevention and control in northern Tanzania. Study findings show that majority of CHWs in northern Tanzania have general knowledge on different NCDs while all of them had favorable attitude toward the burden of NCDs in Tanzania and their involvement as among stakeholders. While more than half of CHWs (63.4%) were found to have been involved themselves in NCDs prevention and control activities, only 26.7% and 46.1% of CHWs were involved in screening activities and had attended NCDs‐related training or seminar, respectively.

More than half of CHWs had good overall knowledge of different NCDs and had been involved in prevention and control activities as it has been reported in several studies done in Uganda [28, 29]. Moreover, most of the CHWs had favorable attitude toward the burden of NCDs in their communities and the role they can play as it was found in previous studies [27, 28]. This portrays interest, willingness, and readiness of CHWs to involve themselves in NCDs prevention and control activities which is a key step in ensuring healthcare for all and sustainability in terms of availability of primary healthcare services at the community level. This will not only enhance early prevention and control of NCDs but also will enable community members to access routine screening, respective health counseling and referrals, community‐based treatment, and palliative care support for different NCDs for free at no cost while at home. Furthermore, this might significantly reduce NCDs economic burden in Tanzania and enhance health‐seeking behaviors such as routine health checkups against NCDs and related complications among community members [13, 14]. On the other hand, the finding that only 46.1% had attended NCDs‐related training contradicts with the fact that the majority had good overall NCDs knowledge. This might be attributed to reporting and self‐desirability bias as this conclusion was based on self‐reported knowledge.

Investing in CHWs as a community‐based strategy for NCD prevention and control is a cost‐effective approach that delivers significant benefits [19, 24, 25, 26]. In India, delivery of home‐based screening for NCDs by trained CHWs was found to be feasible and acceptable [34]. While screening for NCDs and their respective complications is the key in early detection and control of different NCDs, it is only nearly a quarter of CHWs (26.7%) in northern Tanzania have been involved in screening activities. This might be due to the finding that only 36.1% of participants reported to have access to NCDs screening tools.

Despite the fact that majority of CHWs had good overall knowledge of NCDs (92.1%), favorable attitude on NCDs burden and their involvement (100%) and overall practice involvement confidence (69.6%), still on assessing the enabling factors for their involvement in NCDs prevention and control, only less than half (46.1%) reported to have attended a training or seminar on NCDs while 36.1%, 19.9%, and 48.2% reported to have access to tools for NCDs' screening, patient management, and palliative care, respectively. These findings highlight the possible areas of interventions to facilitate the CHWs' involvement in NCD prevention and control.

Regarding challenges faced by CHWs toward their involvement in NCDs prevention and control practice, one study reported poor remuneration as a barrier while another study reported contrary findings [27, 28, 30, 31]. This study found that despite the fact that poor remuneration was reported by majority CHWs as a barrier toward their involvement in NCDs prevention and control practice, but it was not statistically a significant determinant. Most CHWs reported inadequate NCD knowledge, negative community perception toward NCDs as among challenges toward their involvement in NCDs prevention and control as reported in a study in Uganda [28]. Of note, one of the striking findings is that lack of sufficient knowledge of NCD prevention and control was the major challenge acknowledged by 84.3% of the CHWs, however, the overall knowledge was seen to be high which is contrary to the expectations. This discrepancy could be attributed to the reporting and self‐desirability bias credited by the self‐reporting nature of the study.

Among statistically significant determinants of CHWs involvement toward NCDs prevention and control; access to NCDs screening tools, professional training to practice as CHWs, frequency of home visits per week, CHW's involvement confidence, NCDs special training or seminar attendance and access to screening tools were strongly associated with CHWs involvement in NCDs prevention and control activities. This might be due to the fact that all these factors directly enable CHWs and increase the chances of their participation.

### 4.1 Strength and Limitation of the Study

To the best of our knowledge, this is the first study evaluating the role of community healthcare workers in NCD prevention and control in Tanzania, providing valuable insights into their knowledge, attitudes, and involvement. The study was also conducted in areas with the most active CHWs programs in northern Tanzania. This makes the findings more reflective of the status among CHWs in terms of the study aim. Furthermore, it has been conducted amidst the abrupt increase of NCDs cases in Tanzania. The desired sample size was achieved and proper analysis methods were deployed. This has enhanced the precision and power of the study.

On the other hand, self‐reported knowledge and involvement of CHWs in NCD prevention and control might have been subjected to reporting and self‐desirability bias which might have affected the study findings. Supplementing the study findings with a qualitative aspect would strengthen the findings and the conclusion drawn. This has not been done with this study.

## 5 Conclusion and Recommendation

CHWs are useful toward prevention and control of different NCDs. Majority of CHWs showed overall good knowledge, favorable attitude, and high involvement in NCDs prevention and control in northern Tanzania. However, only a few of them were involved in community mobilization and screening of different NCDs. Their limited involvement is attributed secondary to several factors including inability to access NCDs screening tools and lack of training tailored to NCDs–CHWs context.

Several CHWs‐based programs tailored against NCDs should be implemented to maximize CHWs involvement and effectiveness toward NCDs prevention and control in their local communities. Public health stakeholders, including respective government institutions, should design and implement capacity‐building programs among CHWs in terms of trainings for both skills and knowledge acquisition. This should also be incorporated in the national NCDs strategic plans. NCDs training manuals developed in Tanzania for primary healthcare facilities should also be applied toward CHWs. On top of that, these programs should ensure accessibility of tools for screening, patient management, and palliative care among CHWs. Health facilities should work in partnership and harmony locally with CHWs in enabling their involvement toward NCDs prevention and control.

Additionally, professional training to practice as a CHWs should be prioritized in both in service and new enrolled CHWs. This will enhance their knowledge toward their role as CHWs and their involvement in NCDs prevention and control. Since the WHO have made the prevention and control practice guideline (PEN) available to enable CHWs involvement in NCDs prevention and control practice, this guideline should be incorporated among CHWs professional trainings and made available to every CHWs in Swahili format.

## Author Contributions

**Harold L. Mashauri:** conceptualization, data curation, formal analysis, methodology, investigation, project administration, writing – original draft, visualization. **Cornel M. Angolile:** data curation, methodology, investigation, formal analysis, writing – original draft, visualization. **Florida J. Muro:** conceptualization, methodology, investigation, formal analysis, writing – review and editing, supervision. All authors have read and approved the final version of the manuscript.

## Ethics Statement

Ethical approval was sought from Kilimanjaro Christian Medical University College Research and Ethics Review Committee (KCMUCo‐CRERC) with certificate registration number 2610. Permission letters to conduct a study were obtained from local government authorities in Moshi and Arusha Municipals.

## Consent

Written informed consent was obtained from each participant after explaining to them and reading the study information prior to start filling the survey so that they can make an informed decision to participate or withdraw from the study without losing their dignity.

## Conflicts of Interest

The authors declare no conflicts of interest.

## Transparency Statement

The lead author Harold L. Mashauri affirms that this manuscript is an honest, accurate, and transparent account of the study being reported; that no important aspects of the study have been omitted; and that any discrepancies from the study as planned (and, if relevant, registered) have been explained.

## Data Availability

Harold L. Mashauri had full access to all of the data in this study and takes complete responsibility for the integrity of the data and the accuracy of the data analysis. Data collected during this study are available from the corresponding author upon a reasonable request.
